# Phosphorylation of GluA1-Ser831 by CaMKII Activation in the Caudate and Putamen Is Required for Behavioral Sensitization After Challenge Nicotine in Rats

**DOI:** 10.1093/ijnp/pyac034

**Published:** 2022-06-09

**Authors:** Sunghyun Kim, Sumin Sohn, Eun Sang Choe

**Affiliations:** Department of Biological Sciences, Pusan National University, Busan, Republic of Korea; Department of Biological Sciences, Pusan National University, Busan, Republic of Korea; Department of Biological Sciences, Pusan National University, Busan, Republic of Korea

**Keywords:** Glutamate receptor, nicotine, phosphorylation, striatum, tobacco

## Abstract

**Background:**

Phosphorylation of the glutamate receptor (GluA1) subunit of α-amino-3-hydroxy-5-methyl-4-isoxazolepropionic acid (AMPA) receptor plays a crucial role in behavioral sensitization after exposure to psychostimulants. The present study determined the potential role of serine 831 (Ser831) phosphorylation in the GluA1 subunit of the caudate and putamen (CPu) in behavioral sensitization after challenge nicotine.

**Methods:**

Challenge nicotine (0.4 mg/kg) was administered subcutaneously (s.c.) after 7 days of repeated exposure to nicotine (0.4 mg/kg, s.c.) followed by 3 days of withdrawal in rats. Bilateral intra-CPu infusions of drugs were mainly performed to test this hypothesis.

**Results:**

Challenge nicotine increased both phosphorylated (p)Ser831 immunoreactivity (IR) and pCa^2+^/calmodulin-dependentprotein kinases II (pCaMKII)-IR in the medium spiny neurons (MSNs) of the CPu. These increases were prevented by bilateral intra-CPu infusion of the metabotropic glutamate receptor 5 (mGluR5) antagonist MPEP (0.5 nmol/side) and the *N*-methyl-D-aspartate (NMDA) receptor antagonist MK801 (2 nmol/side). However, the dopamine D1 receptor (D1R) antagonist SCH23390 (7.5 nmol/side) prevented only pSer831-IR alone. Bilateral intra-CPu infusion of the Tat-GluA1_D_ peptide (25 pmol/side), which interferes with the binding of pCaMKII to GluA1-Ser831, decreased the challenge nicotine–induced increase in locomotor activity.

**Conclusions:**

These findings suggest that the GluA1-Ser831 phosphorylation in the MSNs of the CPu is required for the challenge nicotine–induced behavioral sensitization in rats. CaMKII activation linked to mGluR5 and NMDA receptors, but not to D1R, is essential for inducing the CaMKII-Ser831 interaction.

**Significance Statement**

Challenge nicotine leads to behavioral sensitization by increasing glutamate release via stimulation of α7 nicotinic acetylcholine receptor (nAChR) in the caudate and putamen (CPu) of rats. However, it is not known how electrochemical changes in the neurons of the CPu result in behavioral sensitization after challenge nicotine. Phosphorylation of α-amino-3-hydroxy-5-methyl-4-isoxazolepropionic acid (AMPA) receptor by Ca^2+^/calmodulin-dependent protein kinase II (CaMKII), which is linked to the stimulation of other glutamate receptors such as the *N*-methyl-D-aspartate (NMDA) receptor and metabotropic glutamate receptor 5 (mGluR5) in the medium spiny neurons of the CPu, is crucial for regulating behavioral sensitization after challenge nicotine. The present study unraveled that GluA1-Ser831 phosphorylation of AMPA receptor by activated CaMKII is required for behavioral sensitization after challenge nicotine. This finding enhances the understanding of neuroadaptation caused by interaction between CaMKII and AMPA receptor on challenge nicotine-induced behavioral sensitization of rats.

## Introduction

The α-amino-3-hydroxy-5-methylisoxazole-4-propionic acid (AMPA) receptor is an ionotropic glutamate receptor consisting of 4 subunits (GluA1-4) (Hollmann and Heinemann, 1994). Stimulation of the AMPA receptor in response to drug exposure upregulates fast ionic conductance. This alters the electrochemical environment of neurons, leading to psychomotor sensitization (Wolf, 1998; Cornish and Kalivas, 2000; Pierce and Wolf, 2013). Intracellular Ca^2+^ mobilization elevated by the stimulation of other glutamate receptors, such as *N*-methyl-D-aspartate (NMDA) receptor and group I metabotropic glutamate receptor (mGluR1/5), after drug exposure further regulates the function of the AMPA receptor (Wang et al., 2005; Oh et al., 2013).

Activation of Ca^2+^-dependent kinases, such as Ca^2+^/calmodulin-dependent protein kinases II (CaMKII) and/or protein kinase C (PKC), which are coupled to glutamate receptors, phosphorylates serine residue at the position of 831 (Ser831) in the C-terminus of GluA1 subunit after cocaine exposure (Roche et al., 1996; Barria et al., 1997; Wang et al., 2006). However, activation of cAMP and/or protein kinase A (PKA), which are coupled to dopamine D1 receptor (D1R), phosphorylates Ser845 in the GluA1 subunit after cocaine exposure (Snyder et al., 2000; Surmeier et al., 2007; Oh et al., 2013). These findings suggest that the psychomotor function of the AMPA receptor is regulated by the phosphorylation of serine residues via activation of protein kinases coupled to glutamate and dopamine receptors in the brain after drug exposure.

Nicotine, a major psychoactive component of tobacco, produces physical and psychological dependence in mammals and human beings (Stolerman and Jarvis, 1995; Le Foll and Goldberg, 2006). Nicotine upregulates glutamate release by stimulating nicotinic acetylcholine receptors (nAChRs) in the terminals of the neurons in the caudate and putamen (CPu), a component of the basal ganglia involving habit learning such as cigarette smoking (Yin and Knowlton, 2006; Changeux, 2010; Ryu et al., 2017). It has been documented that stimulation of α7 nAChR, which is expressed mainly in the glutamate terminals of the CPu, enhances glutamate release after nicotine exposure (Engelman and MacDermott, 2004; Ryu et al., 2017). Challenge exposure to nicotine after withdrawal increases locomotor activity by stimulating α7 nAChR in the CPu of rats (Ryu et al., 2017, 2020), suggesting that elevation of glutamate release leads to behavioral sensitization. Previous studies have demonstrated that increased phosphorylation of Ser831 in the CPu after cocaine exposure causes the elevation of locomotor activity in rats (Martínez-Rivera et al., 2017; Wang et al., 2017; Yang et al., 2018). Taken together, Ser831 in the GluA1 of the AMPA receptor in the neurons of the CPu can be phosphorylated as a result of enhanced glutamate release in response to psychostimulants.

However, it is not known how electrochemical changes in the neurons of the CPu lead to behavioral sensitization in response to challenge nicotine. Therefore, we investigated whether the interaction between CaMKII and Ser831 phosphorylation in the GluA1 of AMPA receptor in the CPu drives behavioral sensitization after challenge nicotine in rats. This study shows a novel finding that the binding of activated CaMKII to GluA1-Ser831 in the γ-aminobutyric acid (GABA) neurons of the CPu is responsible for behavioral sensitization in rats after challenge nicotine.

## MATERIALS AND METHODS

### Animals

Sprague-Dawley male rats (6 weeks, n = 146) were obtained from Hyo-Chang Science Co. (Daegu, Korea). Rats were housed in pairs in a controlled environment with food and water available ad libitum. They were housed in a 12-hour-light/-dark cycle room (light on at 7 am) at a temperature and humidity of 21°C–23°C and 45%–55%, respectively. The rats were allowed to acclimate for a week, and the experiment started when they weighed 250–310 g. Experiments were performed within the light cycle period. On the day of the experiment, injections were given in home cages to minimize stress. All animal procedures were approved by the Institutional Animal Care and Use Committee of Pusan National University and conducted in accordance with the provisions of the NIH Guide for the Care and Use of Laboratory Animals.

### Drugs and Peptides

Nicotine hydrogen tartrate salt was purchased from Sigma-Aldrich (St. Louis, MO, USA), dissolved in sterile 0.9% physiological saline (NaCl), and adjusted to pH 7.2–7.4 with sodium hydroxide. Nicotine was administered subcutaneously (s.c.) in a volume of 1 mL, and the concentration of nicotine (0.4 mg/kg/d) was determined in previous studies (Ryu et al., 2017, 2018). Rats were randomly divided into 2 groups and administered saline or nicotine for 7 consecutive days. After repeated injections followed by a 3-day withdrawal period, the rats received a challenge saline or nicotine injection the day after the withdrawal period. All drugs, except nicotine, were purchased from Tocris Bioscience (Bristol, UK), and solutions were freshly prepared before use. KN62 (CaMKII inhibitor), MPEP (mGluR5 antagonist), MK801 (NMDA receptor antagonist), and SCH23390 (D1R antagonist) were dissolved in the minimum concentration of dimethylsulfoxide and then diluted in artificial cerebrospinal fluid containing (mM) 123 NaCl, 0.86 CaCl_2_, 3.0 KCl, 0.89 MgCl_2_, 0.50 NaH_2_PO_4_, and 0.25 Na_2_HPO_4_ aerated with 95% O_2_/5% CO_2_ (pH 7.2–7.4) or NaCl. The same dimethylsulfoxide-artificial cerebrospinal fluid solution was used as a vehicle control. The concentrations of pharmacological drugs used in this study were determined from previous studies (Ahn and Choe, 2009; Lee et al., 2010; Oh et al., 2013). Two Tat-GluA1-derived peptides (Peptron, Daejeon, South Korea), Tat-GluA1_D_ and Tat-GluA1_A_, contained the Ser831 site of GluA1 in which the Ser831 residue is modified to aspartate (Tat-GluA1_D_, D-peptide) or alanine (Tat-GluA1_A_, A-peptide). D-peptide was synthesized as a competitive inhibitor to compete with phosphorylated (p)Ser831 in the GluA1 subunit (Lee et al., 2013). A-peptide was used as a negative control as described previously (Lee et al., 2013). Both peptides containing the HIV-1 Tat N-terminus domain (YGRKKRRORRR) enhance cell permeability (Schwarze et al., 1999).

### Surgery and Intra-CPu Drug Infusion

The rats were anesthetized with a mixture of Zoletil 50 (tiletamine and zolazepam, 75 μL/kg; Virbac Korea, Seoul) and Rompun (xylazine, 50 µL/kg; Bayer Korea, Seoul), then placed in a stereotaxic apparatus. Under aseptic conditions, a 23-gauge, stainless-steel, double-guide cannula (0.29-mm inner diameter, 13 mm long) was implanted 1 mm anterior to the bregma, 2.5 mm left/right of the midline, and 5 mm below the surface of the skull. The guide cannula was sealed with a stainless-steel wire of the same length. The rats were then allowed 7 days to recover from surgery prior to the experiment. On the day of the experiment, the inner steel wire was replaced with a 30-gauge stainless-steel injection cannula (0.15-mm inner diameter, 13.5 mm long) that protruded 0.5 mm from the guide cannula. Throughout the experiments, all drugs and peptides were infused bilaterally into the central part of the CPu 5 minutes prior to the final injection of saline or nicotine in a volume of 1 μL at a rate of 0.2 μL/min in freely moving rats. The progress of the injection was monitored by observing the movement of a small air bubble along the length of precalibrated PE-10 tubing inserted between the injection cannula and a 2.5-μL Hamilton microsyringe. After the infusion, the injector was left in place for an additional 5 minutes to reduce any possible backflow along the injection tract. The physical accuracy of the injection was verified by the reconstruction of microinjection placements.

### Western-Blot Analysis

Rats were anesthetized with a mixture of Zoletil 50 and Rompun and decapitated 5 minutes after the final injection of saline or nicotine. Brains were then removed, frozen in isopentane at −70°C, and stored in a deep freezer until use. Brain slices were cut serially using a cryostat (Leica Biosystems, Nussloch, Germany) at −20°C, after which both injected sides of CPu were removed using a steel borer (2-mm inner diameter). All tissue samples were sonicated 3 times for 9 seconds in lysis buffer containing (mM) 10 Tris-HCl (pH 7.4), 5 NaF, 1 Na_3_VO_4_, 1 ethylenediaminetetraacetic acid (EDTA), and 1 ethylene glycol-bis(2-aminoethylether)-N,N,N′,N′-tetraacetic acid (EGTA). The lysates were incubated on ice for 1 hour and centrifuged twice at 13 200 rpm for 30 minutes at 4°C. The pellet, which primarily contained nuclei and large debris, was discarded, and the supernatants were used in the experiment. Concentrations of solubilized proteins in the supernatants were determined by the Bradford method using a Bio-Rad Protein Assay (Bio-Rad Laboratories, Hercules, CA, USA). Proteins were resolved by 9%–10% sodium dodecyl sulfate-polyacrylamide gel electrophoresis and transferred to nitrocellulose membranes. The membranes were blocked with 5% skim milk in Tris-buffered saline and Tween-20 (TBST) for 1 hour at room temperature (RT). The membranes were then washed 3 times with TBST. The membranes were probed with anti-pGluA1-Ser831 and Ser845 (Cat. no. 04-823 and 04-1073; rabbit monoclonal; 1:1000; Merck Millipore, Darmstadt, Germany), anti-pCaMKII (Cat. no. MA1-047; mouse monoclonal; 1:500; Thermo Fisher Scientific, Waltham, MA, USA), pPKC (Cat. no. 9375; rabbit polyclonal; 1:2000; Cell Signaling Technology, Danvers, MA, USA), and pPKA (Cat. no. 4781; rabbit polyclonal; 1:2000; Cell Signaling Technology) or anti-GAPDH (Cat. no. 60004-1-Ig; mouse monoclonal; 1:20 000; Proteintech, Chicago, IL, USA) overnight at 4°C on a shaker. Antibodies were diluted with 2% skim milk in TBST. After 3 washes, membranes were incubated with horseradish peroxidase (HRP)-labeled goat anti-rabbit secondary antiserum (1:5000–10 000; KPL, Gaithersburg, MD, USA) or anti-mouse secondary antiserum (polyclonal, 1:10 000; Proteintech) for 1 hour at RT. Images were taken using the iBright CL1000 (Thermo Fisher Scientific) using enhanced chemiluminescence reagents (Ab Frontier, Seoul, South Korea). The membranes were then immersed in stripping buffer (Thermo Fisher Scientific) for 30 minutes at RT and re-probed with anti-GluA1 (Cat. no. 05-855; rabbit monoclonal; 1:2000; Merck Millipore), anti-CaMKII (Cat. no. MA1-048; mouse monoclonal; 1:2000; Thermo Fisher Scientific), or anti-PKC (Cat. no. 2056; rabbit polyclonal; 1:2000; Cell Signaling Technology) and anti-PKA (Cat. no. 4782; rabbit polyclonal; 1:2000; Cell Signaling Technology). Immunoreactive protein bands imaged using the CL1000 were semi-quantified by counting the number of pixels using the NIH Image 1.62 software.

### Immunofluorescence Analysis

Rats were anesthetized with a mixture of Zoletil 50 and Rompun. They were transcardially perfused with ice-cold 4% paraformaldehyde for 7 minutes. The brains were then removed and post-fixed in a 4% paraformaldehyde/10% sucrose for 2 hours at 4°C, followed by phosphate buffered saline (PBS; pH 7.2)/20% sucrose overnight at 4°C (Choe et al., 2004). For tissue preparation, 30-μm-thick coronal slices from CPu were prepared from challenge saline- (n = 4) or challenge nicotine- (n = 4) treated rats using a freezing sliding microtome. Three slices per rat were used for staining. Slices were blocked with a blocking solution containing 0.3% Triton X-100, 5% normal goat serum, and 0.1% bovine serum albumin in PBS (pH 7.4) for 1 hour. The slices were washed with 0.3% Triton X-100 in PBS 3 times and then incubated with the primary antibodies anti-pGluA1-Ser831 (1:500) and anti-pCaMKII (1:500) overnight at 4°C. Antibodies were diluted in 0.1% bovine serum albumin and 0.3% Triton X-100 in PBS. Subsequently, slices were then incubated overnight in a mixture of secondary antibodies containing goat anti-rabbit Alexa Fluor 488 (Cat. no. ab150077; polyclonal; 1:1000; Abcam, Cambridge, MA, USA) and goat anti-mouse Alexa Fluor 594 (Cat. no. ab150116; polyclonal; 1:1000; Abcam) at 4°C. On the next day, after washing 3 times, slices were incubated with Alexa Fluor 405 anti-NeuN (a neuron-specific nuclear protein) antibody (Cat. no. NBP1-92693AF405; mouse monoclonal; 1:1000; Novus Biologicals, Littleton, CO, USA) overnight at 4°C. To avoid cross-reaction between antibodies, anti-NeuN antibody was treated after incubating with the secondary antibodies. Following 3 washes, slices were mounted with aqueous mounting medium (Abcam). Alexa Fluor 594 anti-DARPP-32 antibody (Cat. no. ab214852; rabbit monoclonal; 1:100; Abcam) was used to identify medium spiny neurons (MSNs). Images were taken using a LSM 800 confocal microscope (Carl Zeiss, Jena, Germany) equipped with a 40×/NA 1.2 water immersion lens. The number of neurons and co-localized puncta that co-express pSer831 and pCaMKII in the CPu was analyzed using the image calculator function in ImageJ/Fiji software (NIH, Bethesda, MD, USA) and Zen blue software 3.4 (Carl Zeiss) (Schindelin et al., 2012).

### Behavioral Assessments

Behavioral assessments were conducted as described previously (Ryu et al., 2017, 2018). Rats received a bilateral intra-CPu infusion of D- or A-peptide prior to the final injection of saline or nicotine. After the peptide infusion, locomotor activity was measured in an open field using an infrared photocell-based Opto-Varimex-4 Auto-Track (Columbus Instruments, Columbus, OH, USA) under sound-attenuated and illuminated conditions. Rats were placed in a standard transparent rectangular chamber (44.5 cm × 44.5 cm × 24 cm) and habituated for 30 minutes prior to the experiment to avoid environmental variations. Three pairs of sensors were positioned on orthogonal axes in the cage to provide the coordinates of rat movements in the locomotor testing chamber. Each pair of sensors produced 16 infrared light beams intersecting the animal cage (beam scan rate = 10 Hz). This Auto-Track system sensed the presence of animals by using data generated by infrared beam blocking. Locomotor activities were recorded for over 60 minutes after the final injection of saline or nicotine. The measurements were transferred to a computer using Opto-Varimex-4 Auto-Track Rapid Release software (v4.99B software, Columbus Instruments). All rats were anesthetized with a mixture of Zoletil 50 and Rompun and then brains were removed to identify the placements of guide cannula. One of 48 rats was excluded from the experiment due to health problems after surgery.

### Statistical Analysis

Analysis was conducted using GraphPad Prism 6 software (GraphPad Software Incorporation, San Diego, CA, USA). Results are presented as means ± standard errors, and the statistical significance of differences was accepted for *P* < .05. Intergroup differences between numbers of immunoreactive pixels per measured area determined by western blotting and immunofluorescence were determined using the unpaired *t* test or 2-way ANOVA followed by Tukey’s multiple comparisons test. Statistical significance of differences in total distance travelled between groups was determined by 2-way ANOVA or 2-way mixed ANOVA followed by Tukey’s multiple comparisons test.

## RESULTS

### Challenge Nicotine Increased Phosphorylation of Ser831 and CaMKII in MSNs of CPu

**Figure 1.**
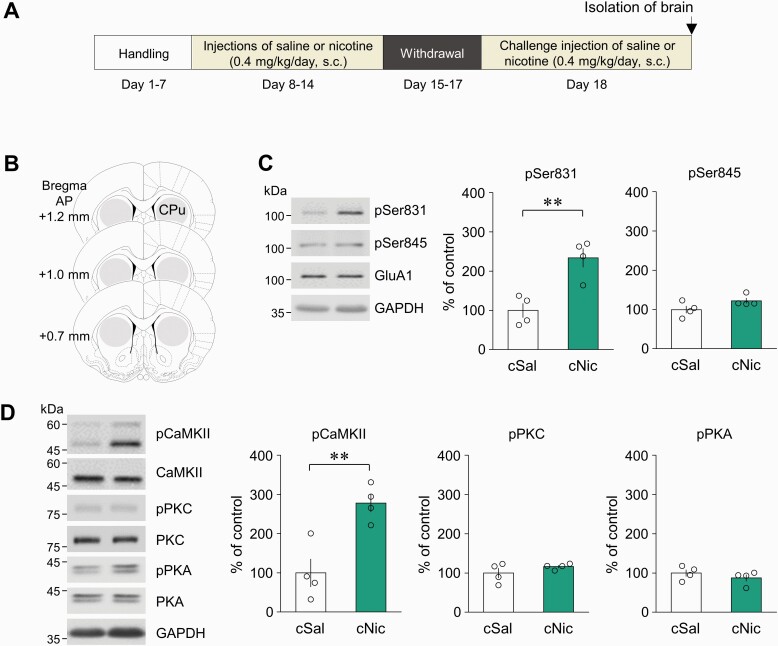
Phosphorylation of Ser831 and protein kinases in the CPu of rats after challenge nicotine. (A) Timeline. (B) Area of the CPu punched out for western blot analysis at the anterior to posterior (AP) of bregma. (C, D) Challenge nicotine (cNic) increased pSer831 and pCaMKII-IR, but not pSer845, pPKC, and PKA-IR compared with challenge saline (cSal). n = 4 rats per group. **P < .01.

**Figure 2.**
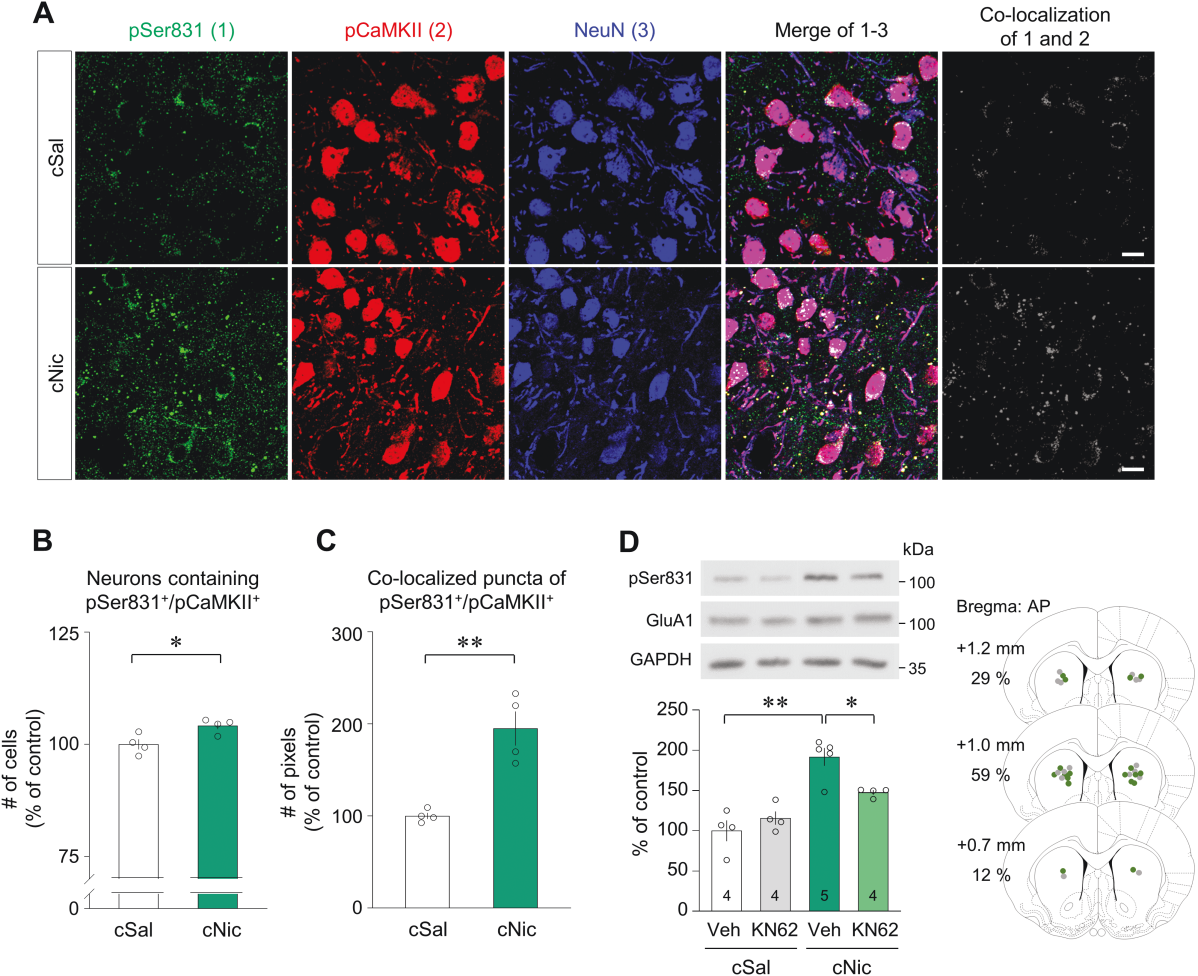
Co-localization of Ser831 and CaMKII phosphorylation in the CPu of rats after challenge nicotine. (A) Triple-immunofluorescence staining for pSer831, pCaMKII, and MSNs in the CPu of rats after challenge exposure to nicotine. Scale bar = 20 μm. n = 4 rats per group. (B, C) Quantitative analysis showed that challenge nicotine increased the number of co-localized neurons and puncta containing pSer831 and pCaMKII in the CPu of rats. (D) Bilateral intra-CPu infusion of the CaMKII inhibitor KN62 decreased the challenge nicotine–induced increase in pSer831-IR. Intra-CPu infusion of KN62 was confirmed by reconstructing microinjection placements (gray dots, cSal groups; green dots, cNic groups). The numbers in the bar graphs refer to the number of rats used in each group. *P < .05; **P < .01.

The first set of experiments was conducted to determine whether challenge nicotine alters Ser831 phosphorylation in the GluA1 subunit of AMPA receptor in the CPu (Fig. 1A, B). Challenge nicotine increased both pSer831 and CaMKII-immunoreactivities (IR), but not pSer845, pPKC, and pPKA-IR compared with those of challenge saline groups (Fig. 1C, D: 1C, *t*(6) = 4.401, *P* = .0046, unpaired *t* test; 1D, *t*(6) = 4.182, *P* = .0058, unpaired *t* test). IR of all measured proteins from the acute nicotine exposure group and the challenge saline group did not show any difference (data not shown). In triple-immunofluorescence staining, challenge nicotine increased the number of neurons, which were co-localized with both pSer831 and pCaMKII-IR that were located mainly in the neurons of the CPu (Fig. 2A, B: 2B, *t*(6) = 2.988, *P* = .0244, unpaired *t* test). Furthermore, >97% of neurons in the CPu were identified as MSNs (supplementary Figure). In addition, the co-localized puncta of pSer831 and pCaMKII-IR were increased within neuronal cell bodies and neuropils (Fig. 2A, C: 2C, *t*(6) = 5.038, *P* = .0024, unpaired *t* test). Because challenge nicotine increased both pSer831 and pCaMKII-IR, the second set of experiments was conducted to determine whether pCaMKII is capable of phosphorylating Ser831 after challenge nicotine. Bilateral intra-CPu infusion of the selective CaMKII inhibitor KN62 (20 nmol/side) attenuated the challenge nicotine–induced increase in pSer831-IR (Fig. 2D: interaction, *F*(1, 13) = 9.061, *P* = .0100; drug effect, *F*(1, 13) = 2.051, *P* = .1758; nicotine effect, *F*(1, 13) = 38.78, *P* < .0001, 2-way ANOVA). The physical accuracy of intra-CPu infusion of KN62 was confirmed by reconstructing microinjection placements (Fig. 2D). Western immunoblotting was conducted at least 3 times throughout the experiments.

### Blockade of mGluR5 and NMDA Receptor Decreased Challenge Nicotine–Induced Increase in Ser831 and CaMKII Phosphorylation

**Figure 3.**
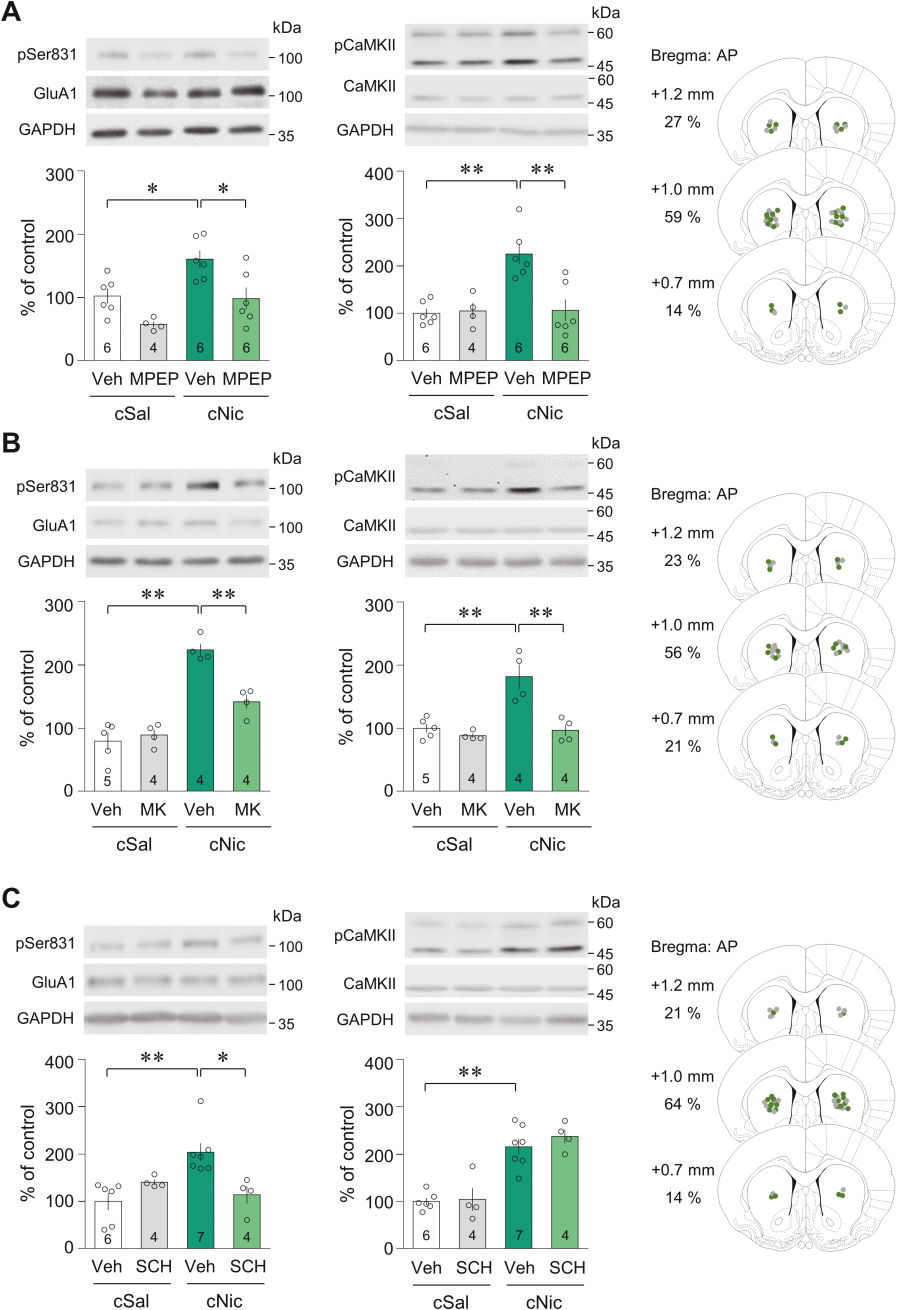
Involvement of glutamate receptors in the phosphorylation of Ser831 by CaMKII activation in the CPu of rats after challenge nicotine. (A, B) Bilateral intra-CPu infusion of the mGluR5 antagonist, MPEP, or NMDA receptor antagonist, MK801 (MK), attenuated the challenge nicotine–induced increase in the pSer831 and pCaMKII-IR. (C) Bilateral intra-CPu infusion of the D1R antagonist, SCH23390 (SCH), decreased elevated pSer831-IR, but not pCaMKII-IR, after challenge exposure to nicotine. Intra-CPu infusion of drugs was confirmed by reconstructing microinjection placements (gray dots, cSal groups; green dots, cNic groups). The numbers in the bar graphs refer to the number of rats used in each group. *P < .05; **P < .01.

Because challenge nicotine increases extracellular glutamate level in the CPu (Ryu et al., 2017), we determined the involvement of glutamate receptors in the phosphorylation of Ser831 and CaMKII. Bilateral intra-CPu infusion of the selective mGluR5 antagonist MPEP (0.5 nmol/side) prior to challenge nicotine decreased the challenge nicotine–induced increase in both pSer831 and pCaMKII-IR (Fig. 3A: pSer831, drug effect, *F*(1,18) = 14.49, *P* = .0013; nicotine effect, *F*(1, 18) = 12.35, *P* = .0025, 2-way ANOVA; pCaMKII, drug effect, *F*(1, 18) = 8.537, *P* = .0091; nicotine effect, *F*(1, 18) = 10.54, *P* = .0045, 2-way ANOVA). Similarly, bilateral intra-CPu infusion of the selective NMDA receptor antagonist MK801 (2 nmol/side) decreased the challenge nicotine–induced increase in both pSer831 and pCaMKII-IR (Fig. 3B: pSer831, drug effect, *F*(1, 13) = 9.927, *P* = .0077; nicotine effect, *F*(1, 13) = 73.40, *P* < .0001, 2-way ANOVA; pCaMKII, drug effect, *F*(1, 13) = 19.09, *P* = .0008; nicotine effect, *F*(1, 13) = 16.71, *P* = .0013, 2-way ANOVA). Challenge nicotine also increased dopamine release by stimulating β2* nicotinic acetylcholine receptors located in the dopamine terminals of the CPu, which are projected from the substantia nigra (Rice and Cragg, 2004). We also determined the involvement of D1R in the phosphorylation of Ser831 and CaMKII. Bilateral intra-CPu infusion of the selective D1R antagonist SCH23390 (7.5 nmol/side) decreased the challenge nicotine–induced increase in pSer831-IR, but not pCaMKII-IR (Fig. 3C: pSer831, interaction, *F*(1, 17) = 12.22, *P* = .0028; drug effect, *F*(1, 17) = 1.773, *P* = .2006; nicotine effect, *F*(1, 17) = 4.336, *P* = .0527, 2-way ANOVA; pCaMKII, drug effect, *F*(1, 17) = 0.6322, *P* = .4375; nicotine effect, *F*(1, 17) = 57.07, *P* < .0001, 2-way ANOVA). However, challenge nicotine did not alter PKA activity, which is coupled to the D1R in the CPu. The physical accuracy of intra-CPu injections was confirmed by reconstructing microinjection placements (Fig. 3A–C).

### Interference of Binding of Activated CaMKII to Ser831 Decreased Challenge Nicotine–Induced Increase in Locomotor Activity in Rats

**Figure 4.**
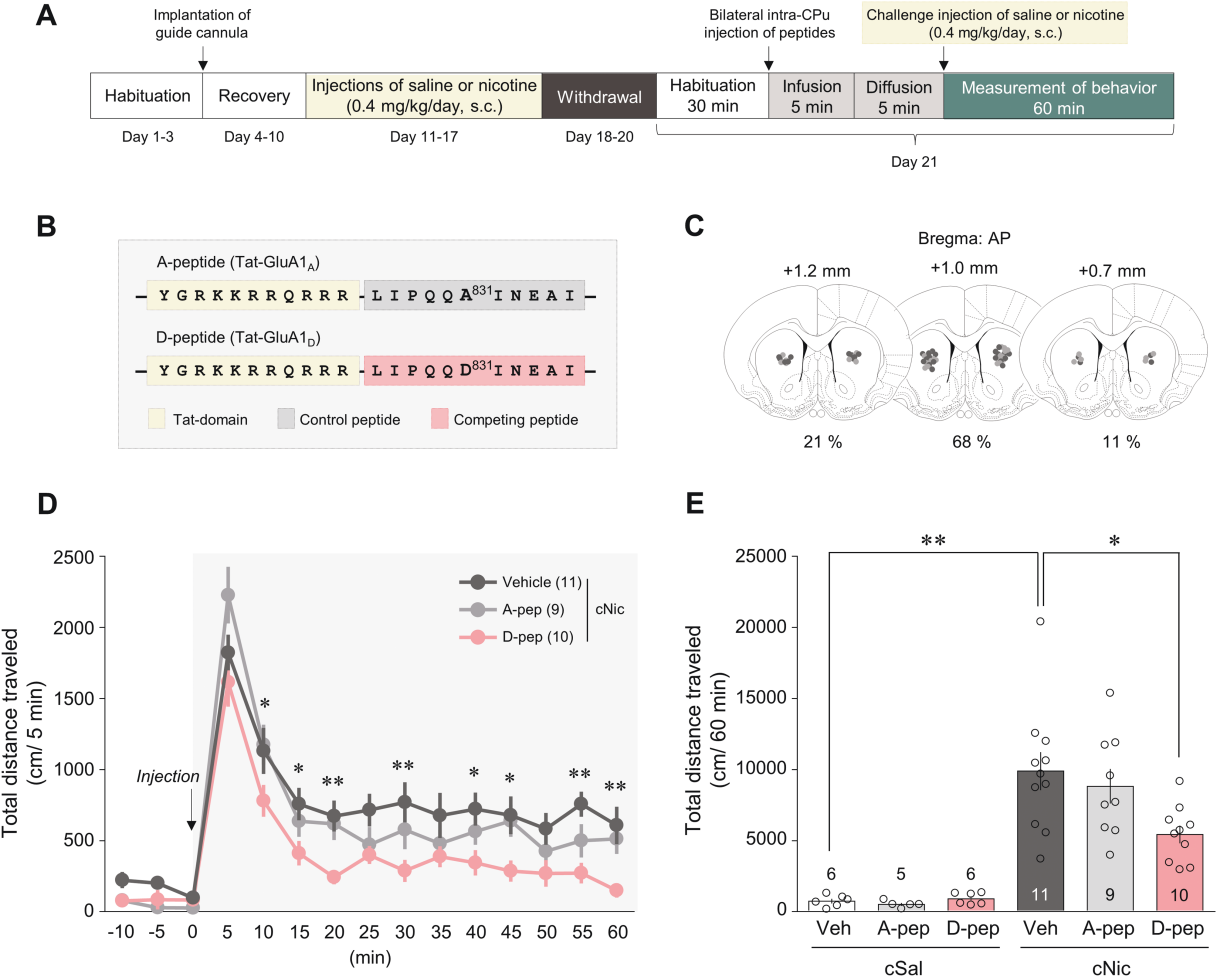
Phosphorylation of Ser831 in the CPu in the regulation of behavioral sensitization in rats after challenge nicotine. (A) Timeline. (B) Amino acid sequences of the 2 synthesized peptides, A- and D-peptide. (C) Intra-CPu infusion of the peptides were confirmed by reconstructing microinjection placements (light gray, challenge saline groups; dark gray, challenge nicotine groups). (D, E) Bilateral intra-CPu infusion of D-peptide decreased the challenge nicotine-induced increase in locomotor activity in rats. D, *P < .05; **P < .01 vehicle vs D-peptide; E, *P < .05; **P < .01. The numbers in the parentheses and bar graphs refer to the number of rats used in each group. A-pep, A-peptide; D-pep, D-peptide.

The contribution of pCaMKII-pSer831 interaction in the behavioral sensitization after challenge nicotine was determined using synthetic peptides (Fig. 4A). D-peptide, which hinders the interaction, was used as a competitive inhibitor of Ser831 of GluA1 subunit, and A-peptide was used as a negative control as described previously (Lee et al., 2013) (Fig. 4B). Reconstruction of microinjection placements demonstrated that the majority of the bilateral intra-CPu infusion of the peptides was confined to the center of the CPu (AP coordinate: +1.2 to approximately +0.7 mm) (Fig. 4C). Bilateral intra-CPu infusion of D-peptide (25 pmol/side) decreased the challenge nicotine–induced increase in total distance travelled over 60 minutes (Fig. 4D–E: 4D, time effect, *F*(14, 378) = 101.9, *P* < .0001; peptide effect, *F*(2, 27) = 4.557, *P* = .0197, 2-way mixed ANOVA; 4E, interaction, *F*(2, 41) = 2.741, *P* = .0763; peptide effect, *F*(2, 41) = 2.199, *P* = .1238; nicotine effect, *F*(1, 41) = 69.88, *P* < .0001, 2-way ANOVA), whereas the infusion of A-peptide (25 pmol/side) did not.

## Discussion

The present study shows that interaction between CaMKII and GluA1-Ser831 in the MSNs of the CPu is necessary for behavioral sensitization after challenge nicotine administration. In the present study, challenge nicotine increased the phosphorylation of Ser831 and CaMKII, but not Ser845, PKC, and PKA phosphorylation in the CPu of rats. Previous studies documented that nicotine upregulates glutamate release in the in vivo recordings of the CPu, resulting in the stimulation of glutamate receptors (Howe et al., 2016; Ryu et al., 2017). Blockade of NMDA receptor and mGluR5 decreased the challenge or chronic nicotine-induced increase in behavioral sensitization in rats (Shoaib et al., 1997; Shim et al., 2002; Paterson et al., 2003). Stimulation of NMDA receptor and mGluR5 increases intracellular Ca^2+^ mobilization either by itself or by interacting with them (Kotecha et al., 2003). This increase in turn activates the Ca^2+^-dependent protein kinases, CaMKII and/or PKC, leading to phosphorylation of GluA1-Ser831 (Kim et al., 2009). Parallel to glutamate release, nicotine upregulates dopamine release, stimulating D1R in the CPu of rats (Shim et al., 2001; Janhunen et al., 2005). Activation of cAMP and/or PKA, which are coupled to D1R, preferentially phosphorylate Ser845 rather than Ser831 in the GluA1 (Snyder et al., 2000; Ahn and Choe, 2009). Taken together, these findings suggest that challenge exposure to nicotine phosphorylates GluA1-Ser831 by activating CaMKII rather than PKC, which is linked to the NMDA receptor and mGluR5 in the CPu.

The present data obtained from triple immunofluorescence staining show that phosphorylation of Ser831 and CaMKII is co-localized in the neurons of the CPu. Furthermore, the co-localized cluster of phosphorylated Ser831 and CaMKII is observed in cell bodies as well as dendrites. It is well-known that medium spiny GABA neurons, which project to the globus pallidus, constitute >90% of the CPu (Kemp and Powell, 1971; Märtin et al., 2019). Consistently, the present study also demonstrates that >97% of neurons in the CPu are overlapped with the MSN marker DARPP-32. These findings support the fact that challenge nicotine phosphorylates GluA1-Ser831 by activating CaMKII in the GABA neurons of the CPu. This notion can be supported by the present finding that inhibition of CaMKII partially reduces the challenge nicotine–induced increase in Ser831 phosphorylation in the MSNs of the CPu.

The present data show that blockade of mGluR5, a dominant group I mGluR subtype in the CPu, reduces the challenge nicotine–induced increase in the phosphorylation of Ser831 and CaMKII. This finding suggests that stimulation of mGluR5 after challenge nicotine phosphorylates Ser831 by activating CaMKII. It is well-documented that stimulation of phospholipase C (PLC) coupled to mGluR5 upregulates the hydrolysis of phosphatidylinositol 4,5-bisphosphate to diacylglycerol (DAG) and inositol 1,4,5-trisphosphate (IP_3_) (Nishizuka, 1984; Abe et al., 1992). Activation of DAG in turn activates PKC, which leads to Ca^2+^ influx by phosphorylating NMDA receptor and/or autophosphorylating CaMKII (Tingley et al., 1997; Liao et al., 2001; Bayer et al., 2001; Giese, 2021). In parallel with PKC activity, elevation of IP_3_ further increases intracellular Ca^2+^ release from the endoplasmic reticulum, resulting in CaMKII activation (Berridge and Irvine, 1984; Yang et al., 2021). In this study, blockade of ionotropic NMDA glutamate receptor reduced the phosphorylation of Ser831 and CaMKII, which were elevated by challenge exposure to nicotine. Taken together, these findings suggest that elevation of Ca^2+^ mobilization, resulting from the stimulation of NMDA receptor and mGluR5, or possibly by an interaction between these 2 receptors, activates CaMKII and phosphorylates GluA1-Ser831 in the MSNs of the CPu. The present data also show that blockade of D1R decreased the challenge nicotine–induced increase in Ser831 phosphorylation, but not CaMKII phosphorylation. Stimulation of D1R after challenge nicotine also contributes to Ser831 phosphorylation by phosphorylating NMDA receptors (Dudman et al., 2003; Chen et al., 2004). Taken together, the present finding suggests that activation of CaMKII due to glutamate receptor stimulation after challenge nicotine is crucial for GluA1-Ser831 phosphorylation in the MSNs of the CPu.

D-peptide that is designed to interfere with the binding of activated CaMKII to Ser831 attenuated the challenge nicotine–induced increase in locomotor activity in rats in this study. Previous studies have demonstrated that Ser831 phosphorylation is necessary for cocaine-induced locomotor sensitization in rats (Martínez-Rivera et al., 2017; Yang et al., 2018). Prevention of Ser831 phosphorylation by inhibiting CaMKII decreases the α7 nAChR-stimulated upregulation of AMPA currents in the rat prefrontal cortex (Tang et al., 2015). Taken together, the present findings suggest that the interaction between activated CaMKII and Ser831 is necessary for behavioral sensitization after challenge nicotine in rats.

**Figure 5.**
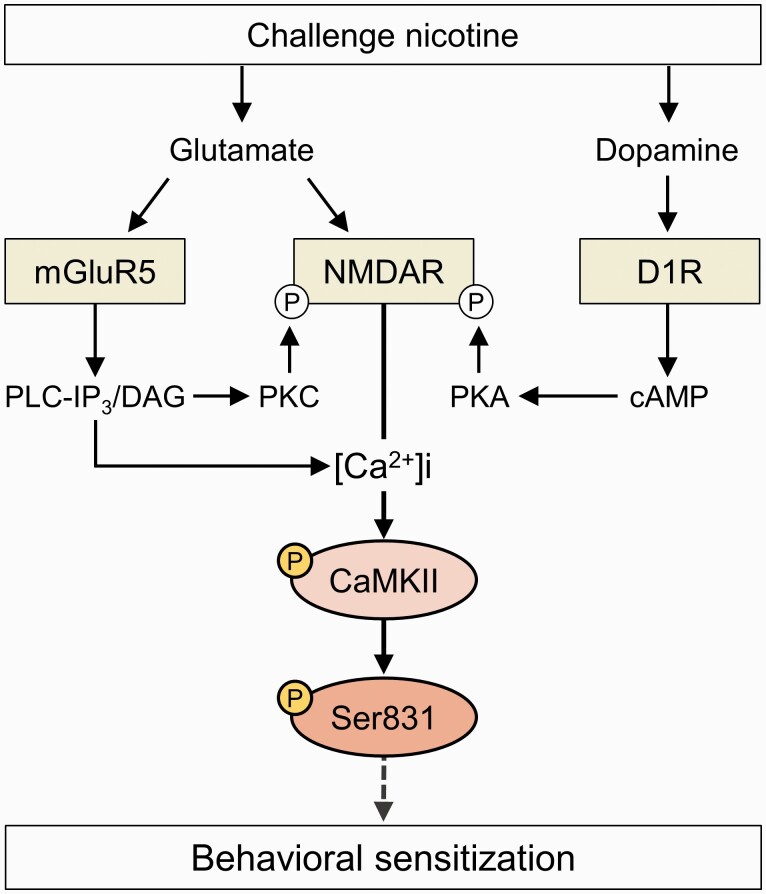
A proposed diagram illustrating molecular changes underlying the challenge nicotine–induced behavioral sensitization via AMPA GluA1-Ser831 phosphorylation in the MSNs of the CPu. Putative interactions are discussed in detail in the text. Elevation of glutamate release after challenge nicotine stimulates mGluR5 and NMDAR, leading to CaMKII phosphorylation by enhancing Ca^2+^ influx and efflux from NMDAR and ER, respectively. In the meantime, stimulation of D1R after challenge nicotine also results in the phosphorylation of Ser831, probably by phosphorylating NMDAR. Phosphorylated CaMKII then phosphorylates GluA1-Ser831, leading to behavioral sensitization in rats. Solid and broken arrows represent direct and indirect stimulation of the downstream molecules, respectively. [Ca^2+^]i, intracellular calcium concentration; P, phosphorylation.

In summary, as proposed in Figure 5, increased glutamate release after challenge nicotine stimulates mGluR5 and NMDA receptors in the CPu, which leads to CaMKII activation by enhancing intracellular Ca^2+^ mobilization. Stimulation of D1R after challenge nicotine also results in the phosphorylation of Ser831 without activating CaMKII. Activated CaMKII in the MSNs of the CPu binds directly to Ser831 in the C-terminus of GluA1, leading to behavioral sensitization. Therefore, the phosphorylation of GluA1-Ser831 by activated CaMKII in the MSNs of the CPu may be necessary for behavioral sensitization in rats after challenge exposure to nicotine. Under these circumstances, stimulation of mGluR5 and NMDA receptor, but not D1R, is linked to CaMKII activation.

## Supplementary Material

pyac034_suppl_Supplementary_Figure
