# Supplementary material for: Phosphorylation of GluA1-Ser831 by CaMKII Activation in the Caudate and Putamen Is Required for Behavioral Sensitization After Challenge Nicotine in Rats
Source: Int J Neuropsychopharmacol. 2022 Jun 9;25(8):678–87. doi: 10.1093/ijnp/pyac034 (PMC9380710; doi:10.1093/ijnp/pyac034)
Supplement: pyac034_suppl_Supplementary_Figure [file pyac034_suppl_supplementary_figure.docx]

**Supplementary Figure**


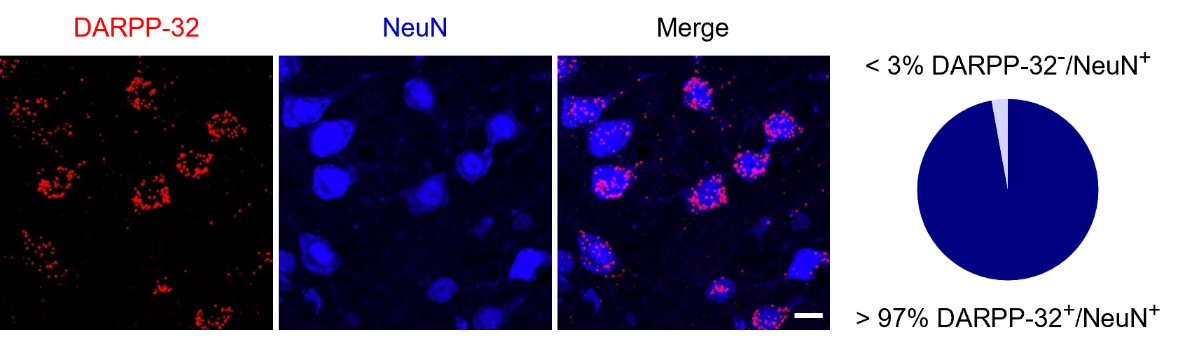


Co-localization of DARPP-32 and NeuN in the CPu of rats using immunofluorescence demonstrated that over 97% of neurons is identified as medium spiny neurons. Scale bar = 20 μm.
